# Midkine derived from cancer-associated fibroblasts promotes cisplatin-resistance via up-regulation of the expression of lncRNA ANRIL in tumour cells

**DOI:** 10.1038/s41598-017-13431-y

**Published:** 2017-11-24

**Authors:** Dongya Zhang, Liang Ding, Yi Li, Jing Ren, Guoping Shi, Yong Wang, Shuli Zhao, Yanhong Ni, Yayi Hou

**Affiliations:** 1 The State Key Laboratory of Pharmaceutical Biotechnology, Division of Immunology and Hospital of Stomatology, Medical School, Nanjing University, Nanjing, China; 2 Nanjing First Hospital, Nanjing Medical University, Nanjing, China

## Abstract

Midkine (MK) is a heparin-binding growth factor that promotes carcinogenesis and chemoresistance. The tumour microenvironment (TME) can affect chemotherapy sensitivity. However, the role of stromal-derived MK, especially in cancer-associated fibroblasts (CAFs), is unclear. Here, we confirmed that MK decreased cisplatin-induced cell death in oral squamous cell carcinoma (OSCC) cells, ovarian cancer cells and lung cancer cells. We also isolated primary CAFs (n = 3) from OSCC patients and found that CAFs secreted increased levels of MK, which abrogated cisplatin-induced cell death. Moreover, MK increased the expression of lncRNA ANRIL in the tumour cells. Normal tissues, matched tumour-adjacent tissues and OSCC tissues were analysed (n = 60) and showed that lncRNA ANRIL was indeed overexpressed during carcinogenesis and correlated with both high TNM stage and lymph node metastasis (LNM). Furthermore, lncRNA ANRIL knockdown in tumour cells inhibited proliferation, induced apoptosis and increased cisplatin cytotoxicity of the tumour cells via impairment of the drug transporters MRP1 and ABCC2, which could be restored by treatment with human MK in a caspase-3/BCL-2-dependent manner. In conclusion, we firstly describe that CAFs in the TME contribute to the high level of MK in tumours and that CAF-derived MK can promote cisplatin resistance via the elevated expression of lncRNA ANRIL.

## Introduction

As an alkylating agent, cisplatin (cis-diamminedichloroplatinum, DDP), is one of the most effective and commonly used chemotherapeutic agents for oral squamous cell carcinoma (OSCC) and other solid tumours, including testicular, ovarian, cervical and non-small-cell lung cancer^1^. However, although cisplatin is very effective in the treatment of these tumours, the cancer cells often possess intrinsic or acquired resistance against chemotherapeutic drugs^2^, which is a considerable obstacle to the successful clinical application of cisplatin in OSCC and other cancers.

Midkine (MK) is a member of the heparin-binding growth factor or cytokine family, which includes pleiotrophin^3^. In recent years, a large number of studies have demonstrated higher expression of MK in the majority of malignant tissues, such as in oral, gastrointestinal, hepatobiliary, lung, ovarian, and prostate cancers^4^, than that expressed in adjacent normal tissues. It has been reported that MK promotes tumour progression by enhancing carcinoma cell growth and survival^5,6^, cell invasiveness and migration and chemotherapy resistance^7–11^. Previously, we found that MK plays a potential role in tumourigenesis. MK inhibits the cytotoxicity of NK cells via increasing the expression of MICA/B and CHOP via the P38-MAPK signalling pathway^12^. Additionally, MK renders glioma cells resistant to tetrahydrocannabinol (THC) by blocking the ALK receptor and inhibiting the activation of autophagy-mediated cell death by the Akt/mTORC1 pathway^13^. However, all these studies focused merely on tumour-derived MK in an autocrine manner; the role of stroma-derived MK still needed to be clarified.

The interplay between stromal cells and tumour cells plays a major role in tumour progression. Cancer-associated fibroblasts (CAFs), which constitute most stromal cells in cancer tissues, secrete a wide spectrum of chemokines and cytokines to the tumour microenvironment, thus promoting the growth, invasion and angiogenesis of cancers^14–16^. The presence of CAFs is correlated with tumour development and worse prognosis of cancer patients, which indicates that CAFs are involved in chemotherapy resistance^17,18^. More recently, emerging evidence indicates that CAFs are involved in chemotherapy resistance. The co-culture of CAFs and oesophageal squamous cell carcinoma (OSCC) cells promotes increased expression and activation of FOXO1 and results in a TGFβ1 autocrine/paracrine signalling loop. Finally, the OSCC cells enhance chemotherapy resistance^19^. Therefore, we speculated that CAF-derived MK could promote chemotherapy resistance.

Currently, lncRNAs are simply classified as transcripts longer than 200 nucleotides with unapparent coding potential, similar to most mRNAs^20^. More recently, numerous lncRNAs have been identified to be closely related to the progression of human cancers^21^. The antisense non-coding RNA at the INK4 locus (ANRIL) is transcribed as a 3834-nt lncRNA that contains 19 exons in the antisense direction of the INK4b-ARF-INK4 gene clusters, which encode three important tumour suppressor genes, p14ARF, p15INK4b and p16INK4a^22^. ANRIL is regarded as a risk factor in tumourigenesis^23,24^. For instance, overexpression of lncRNA ANRIL in prostate cancer was involved in the cis-repression of the p16/ARF gene cluster by directly binding to PRC1 via CBX7^25^. Another study suggested that overexpression of lncRNA ANRIL was closely associated with the poor prognosis of patients with NSCLC and enhanced cell proliferation and apoptosis by binding to PRC2 to induce epigenetic silencing of KLF2 and P21 transcription^26^. However, the effects of lncRNA ANRIL on chemoresistance are still not well understood.

Our current study aimed to investigate the relationship between CAF-derived midkine, lncRNA ANRIL and chemoresistance. Here, our study showed that CAFs could secrete more MK than tumour cells to abrogate cisplatin-induced cell apoptosis. lncRNA ANRIL was up-regulated in cancer cells with CAF supernatant or recombinant human MK (rMK) treatment, which promoted drug resistance. We are the first to find that CAF-derived MK promoted cisplatin resistance via elevated expression of lncRNA ANRIL, which could be regarded as a novel therapeutic target for cancer.

## Results

### Midkine promotes tumour cell resistance to cisplatin

**Figure 1:**
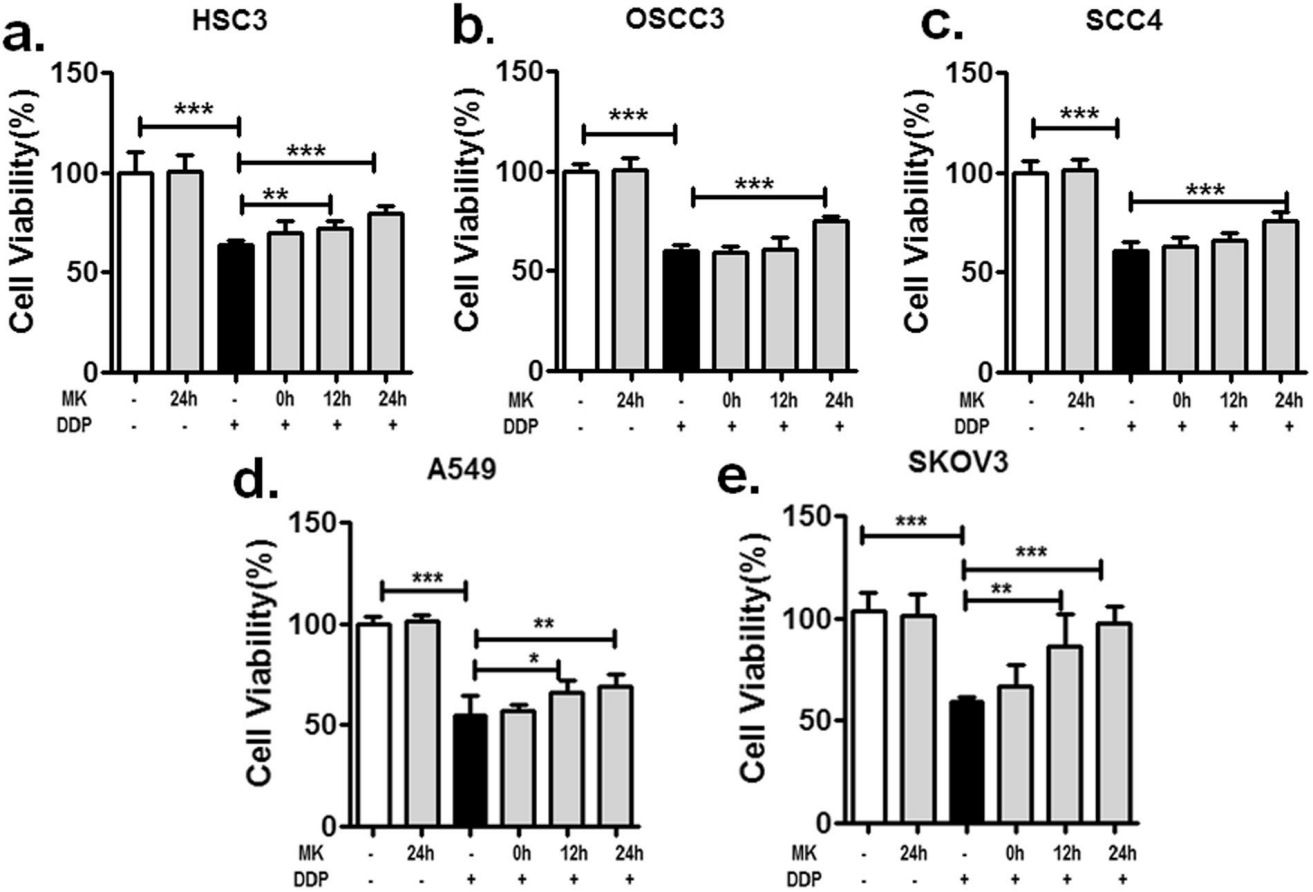
MK increases the survival of cisplatin-treated tumour cells *in vitro*. The effect of cisplatin on tumour cells with MK pretreatment was determined by means of cell viability assays via a CCK-8 kit (*P < *0*.*05*, **P < *0*.*01*, ***P < *0*.*001*). HSC3 (**a**), OSCC3 (**b**), SCC4 (**c**), A549 (**d**) and SKOV3 (**e**) cells were treated with exogenous MK (100 ng/mL) for the indicated time before treatment with the corresponding concentrations of cisplatin (HSC3 6 µM, OSCC3 5 µM, SCC4 9 µM, A549 4.6 µM, and SKOV3 12 µM) for 48 h. Data are representative of three independent experiments.

To determine whether MK-enhanced tumour cells are resistant to cisplatin, we first determined the IC50 values of several tumour cells, including the human oral squamous cell lines HSC3, OSCC3 and SCC4; the human ovarian cancer cell line A2780; and the lung cancer cell line A549 based on their differing sensitivities to cisplatin-induced cell death. The IC50 values of cisplatin for these tumour cell lines were 6 µM, 5 µM, 9 µM (as shown in our previous research^27^), 7.5 µM and 4.6 µM (Supplementary Fig. S1). Then, the cell viability of tumour cells treated with MK for various times before 48 h treatment with the corresponding IC50 level of cisplatin was measured by CCK-8 assay. We found that the cell viability of HSC3, OSCC3 and SCC4 cells with MK treatment for 24 h was increased compared to cells without MK treatment (Fig. 1a–c); the results of experiments using A2780 and A549 cells were similarly increased (Fig. 1d,e). In addition, we discovered that MK did not obviously enhance the survival of tumour cells treated with only MK compared with that of untreated control cells. These observations strongly support that MK is correlated with resistance to cisplatin-induced cell death.

### CAFs in tumour stroma contribute to MK-related cisplatin resistance

**Figure 2:**
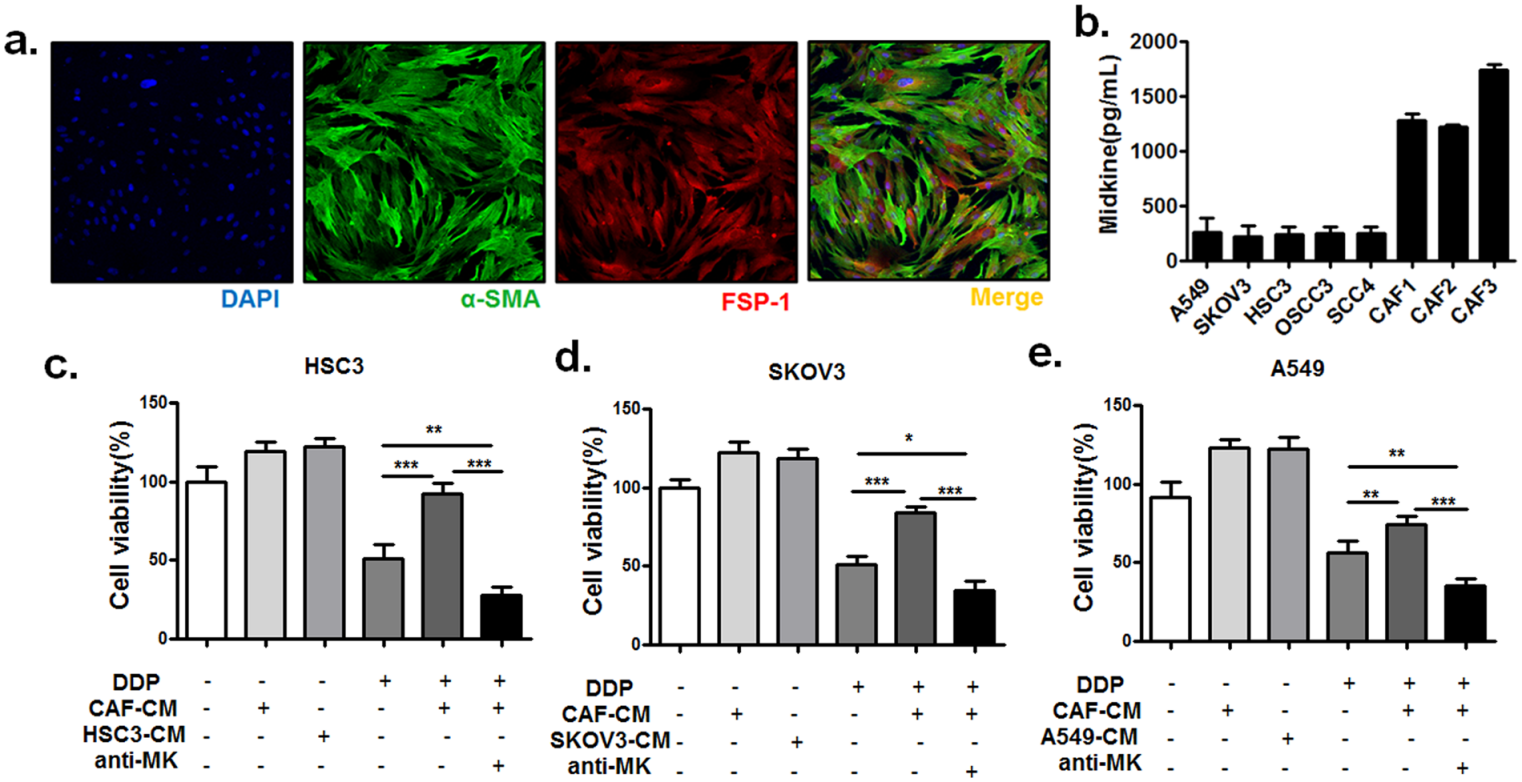
CAF-derived MK promotes tumour cell resistance to cisplatin. (**a**) CAFs were isolated from three OSCC patients and cultured. Phenotyping of the cells was performed by staining for the CAF markers α-SMA and FSP-1 via IF. (**b**) The level of MK in the supernatant of OSCC cell lines (HSC3, OSCC3, SCC4), A549 cells, SKOV3 cells and CAFs that had been cultured for 7 days were determined by ELISA kit. The effect of cisplatin on the viability of HSC3 (**c**), SKOV3 (**d**) and A549 (**e**) cells incubated with either conditioned medium (CM) obtained from CAFs alone or with conditioned medium and a neutralizing anti-MK antibody. The conditioned medium obtained from corresponding tumour cells was used as a positive control (*P < *0*.*05*, **P < *0*.*001*, ***P < *0*.*001*). Representative data are shown from 3 independent experiments.

There have been reports showing that CAFs can secrete a wide spectrum of cytokines. We hypothesized that CAFs would be able to secrete MK and that conditioned medium derived from CAFs would be able to promote tumour cell resistance to cisplatin. To verify this possibility, we isolated primary CAFs from OSCC tissues based on their expression of well-known CAF markers a-smooth muscle actin (a-SMA) and fibroblast-specific protein-1 (FSP-1) (Fig. 2a)^28^. Then, we incubated OSCC HSC3 cells, human ovarian cancer A2780 cells and lung cancer A549 cells in conditioned medium from primary CAFs. In the conditioned medium from CAFs, we found a greater increase in MK than in tumour cells as determined by a human MK ELISA kit (Fig. 2b). As shown in Fig. 2c, we found that incubation with CAF-conditioned medium rendered OSCC cells resistant to cisplatin treatment, an effect that was abrogated by MK neutralization with an anti-MK antibody. These observations strongly support that MK is a factor in resistance to cisplatin-induced OSCC cell death. Altogether, these results suggest that MK is released from CAFs via paracrine secretion and that it promotes tumour cell resistance to cisplatin through inhibition of the cell apoptosis in the tumour microenvironment.

### The expression of lncRNA ANRIL is up-regulated by CAF-derived MK in tumours

**Figure 3:**
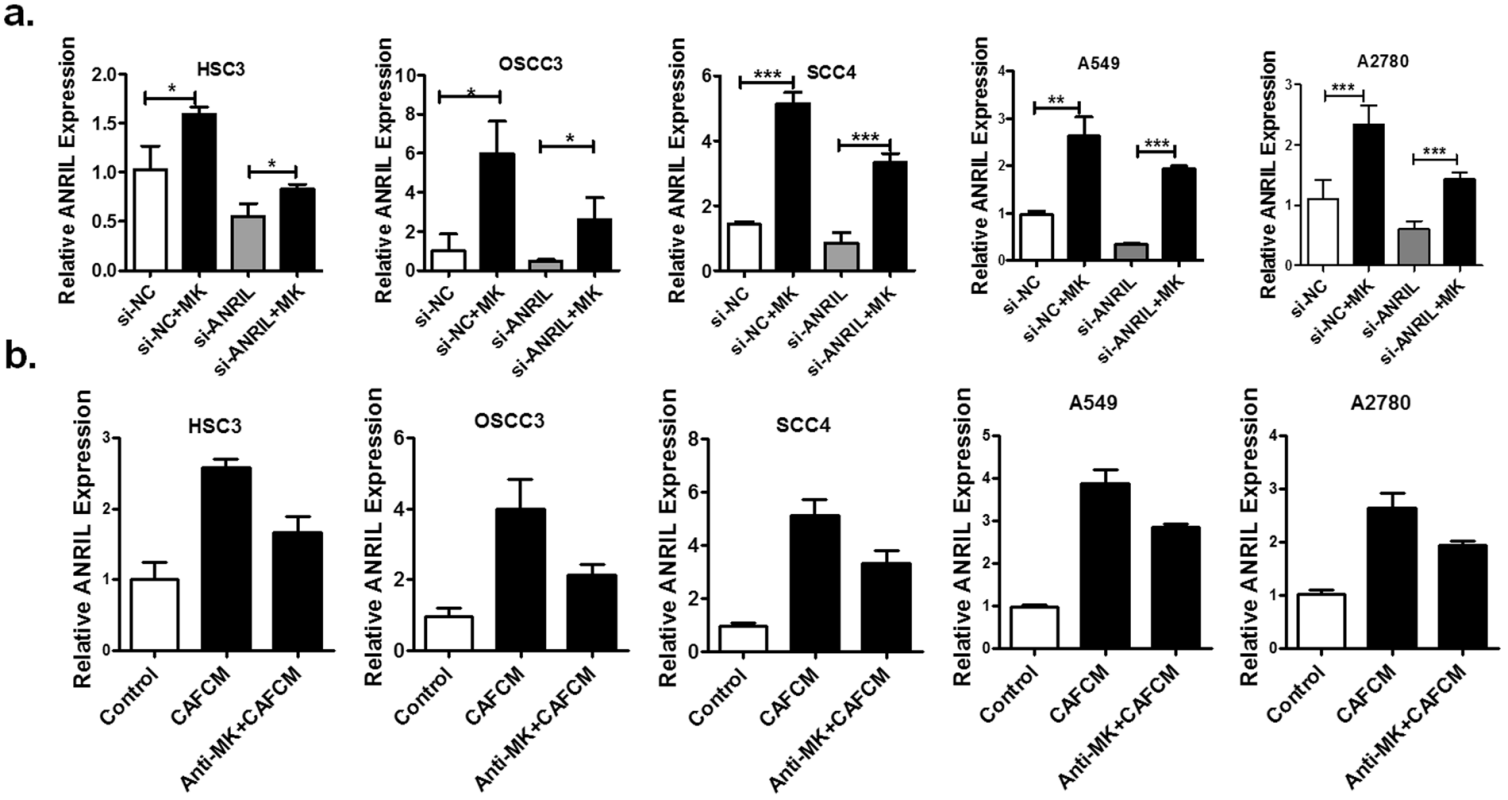
MK increases the expression of ANRIL in tumour cells *in vitro*. (**a**) The level of ANRIL expression in human OSCC cell lines (HSC3, OSCC3, and SCC4), A549 cells and SKOV3 cells transfected with negative control (si-NC) or si-ANRIL for 48 h before treatment with MK (100 ng/mL) for 24 h was determined by qPCR. (**b**) The level of ANRIL expression in human OSCC cell lines, A549 and SKOV3 cells transfected with negative control (si-NC) or si-ANRIL for 48 h before treatment with conditioned medium obtained from CAFs for 24 h was determined by qPCR. Values represent the mean ± SD from three independent experiments (*P < *0*.*05*, **P < *0*.*01*, ***P < *0*.*001*).

As mentioned above, we postulated that MK could induce tumour cell resistance to cisplatin by regulation of lncRNA ANRIL expression. To validate our assumption, we transfected 3 human OSCC cell lines, the lung cancer cell line A549 and the human ovarian cancer cell line A2780 with si-ANRIL or si-NC before treatment with MK (100 ng/mL) and used qPCR analysis to examine the expression of lncRNA ANRIL. As shown in Fig. 3, the level of lncRNA ANRIL expression in tumour cells transfected with si-ANRIL or in the treatment group transfected with si-NC was markedly increased after stimulation with MK for 24 h. Furthermore, lncRNA ANRIL expression was significantly up-regulated in tumour cells that had been stimulated with CAF-conditioned medium for 24 h. In addition, ANRIL expression was obviously decreased in the treatment group with an anti-MK antibody compared with that in the control group. These results illustrate that CAF-derived MK can enhance OSCC cell resistance to cisplatin by up-regulating the expression of lncRNA ANRIL.

### Overexpressed lncRNA ANRIL in OSCC correlates with the poor clinical outcome of OSCC patients

**Figure 4:**
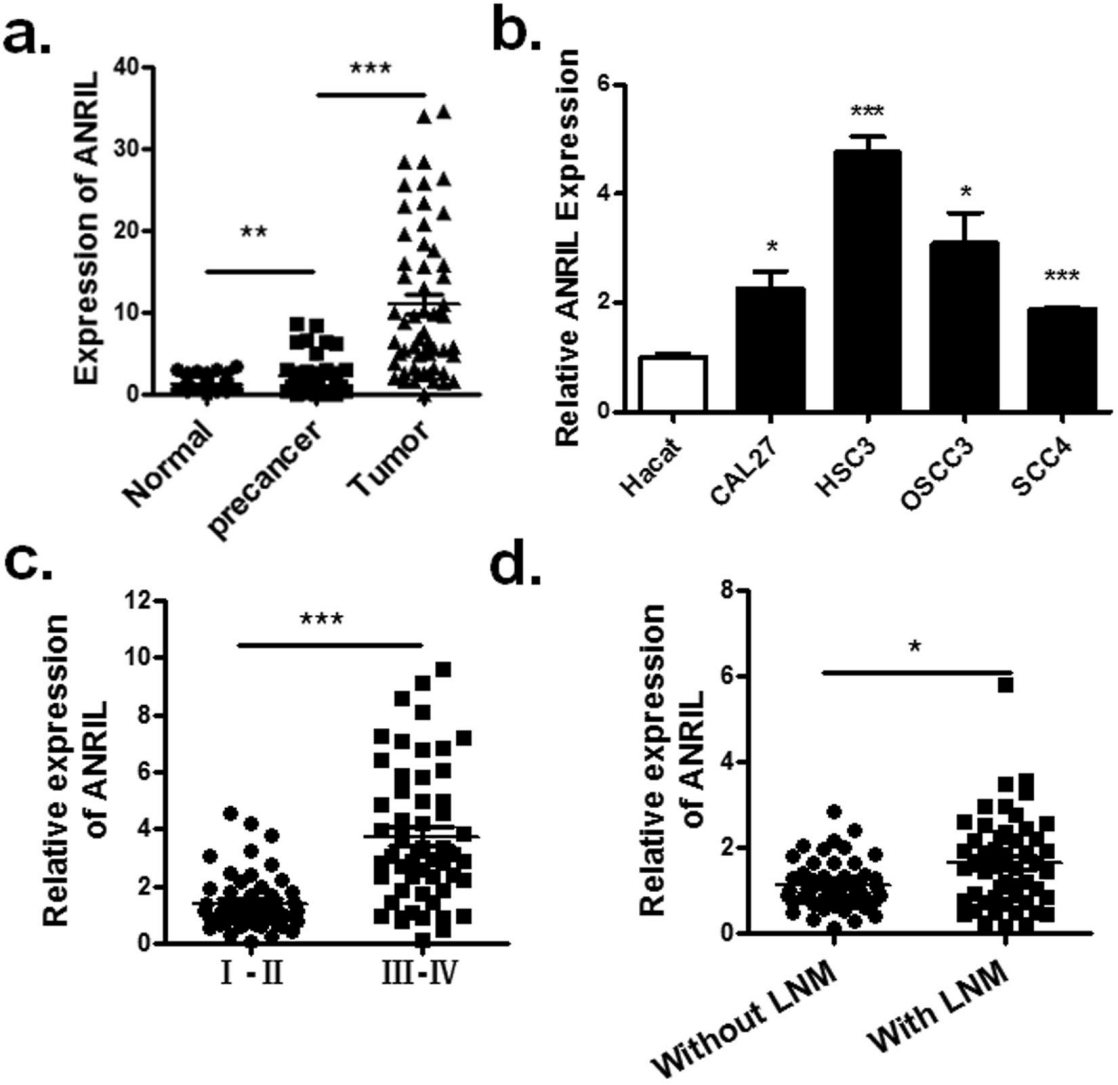
Relative ANRIL expression levels and their association with poor clinical outcome in OSCC tissues. (**a**) Relative ANRIL expression levels in paired OSCC samples were detected by qPCR. (**b**) Related ANRIL expression levels of human oral squamous cancer cell OSCC3, SCC4, HSC3 and CAL27 compared with that in human immortal keratinocyte line HaCaT. The association between the expression of ANRIL in tumour samples and TNM (**c**) or lymph node metastasis (**d**) was analysed (*P < *0*.*05*, **P < *0*.*01*, ***P < *0*.*001*).

To investigate the potential associations between lncRNA ANRIL expression and patient clinicopathological features, we collected a cohort including 60 normal oral tissues, 60 precancer tissues, and 60 OSCC samples for estimating the expression of lncRNA ANRIL in OSCC. We found that lncRNA ANRIL was significantly up-regulated in tumour tissues compared with their normal counterparts (P < 0.01) (Fig. 4a). In addition, we performed qRT-PCR to evaluate the levels of lncRNA ANRIL in four OSCC cell lines and one normal human keratinocyte cell line (HaCaT). In line with the results from the tumour tissues, the expression of lncRNA ANRIL was higher in all four cancer cell lines compared with the levels observed in HaCaT cells, with the highest expression in HSC3 cells (Fig. 4b). Furthermore, we analysed the correlations between aberrant lncRNA ANRIL expression and the clinicopathological characteristics of OSCC patients. The results showed that the expression of lncRNA ANRIL was significantly higher in patients with higher tumour node metastasis (TNM) stage and lymph node metastasis (Fig. 4c,d).

### Aberrant expression of lncRNA ANRIL enhances cell proliferation and cisplatin resistance

**Figure 5:**
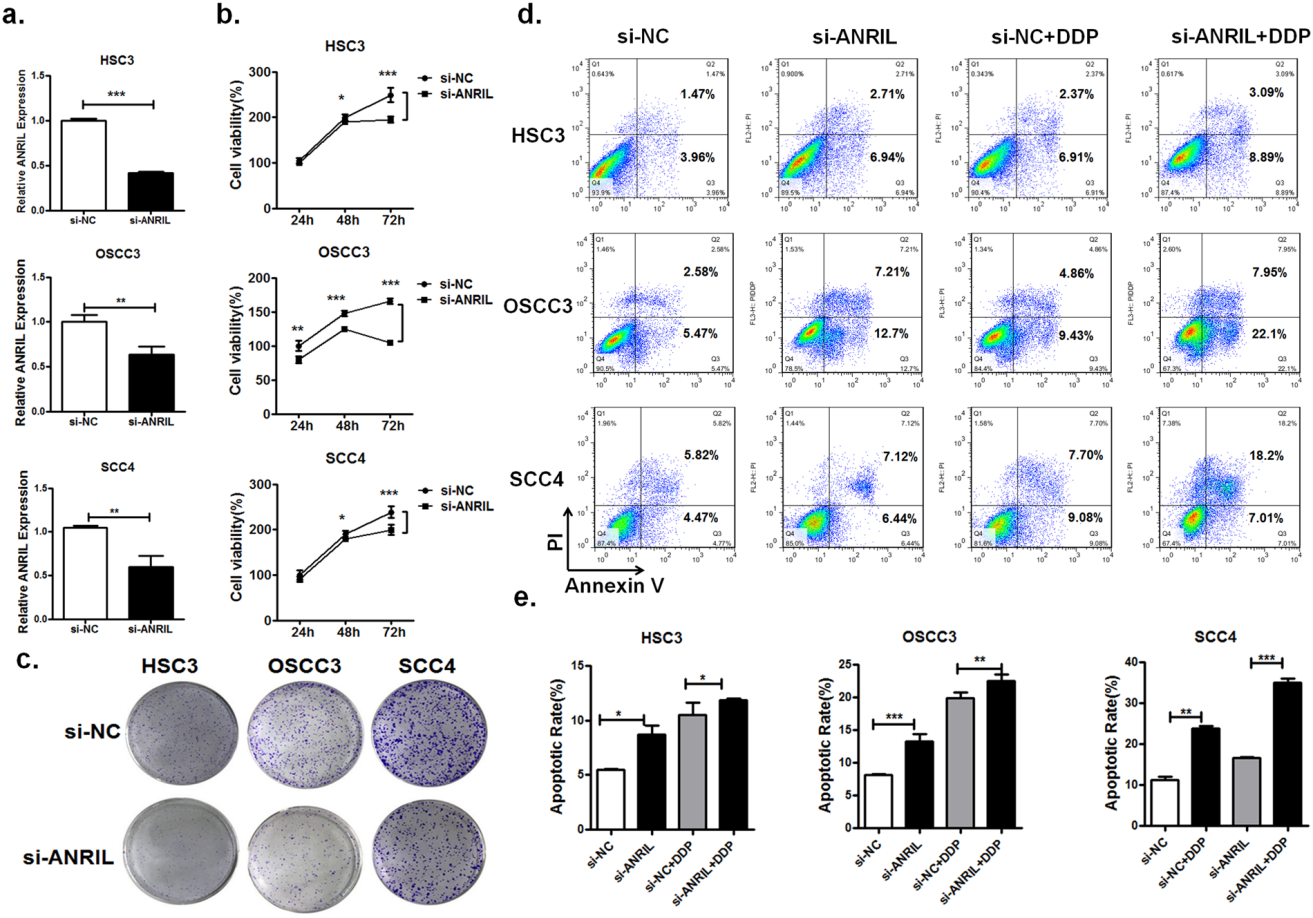
Silencing of ANRIL in OSCC cells inhibits cell growth and reduces OSCC cell resistance to cisplatin *in vitro*. (**a**) Negative control (si-NC) or si-ANRIL was transfected into in HSC3, OSCC3 and SCC4 cells, and they were incubated for 48 h. The level of ANRIL expression was measured by Q-PCR (*P < *0*.*05*, **P < *0*.*01*, ***P < *0*.*001*). (**b**) Knockdown of ARNIL significantly suppressed cell proliferation for the indicated amount of time. Cell viability was determined by CCK-8 assays (*P < *0*.*05*, **P < *0*.*01*, ***P < *0*.*001*). (**c**) OSCC cell lines transfected with si-ANRIL decreased the number of clones compared to si-NC-transfected OSCC cells. The cisplatin-induced apoptosis of OSCC cells transfected with either si-NC or si-ANRIL was analysed by flow cytometric assays (**d**) and quantified (**e**) (*P < *0*.*05*, **P < *0*.*01*, ***P < *0*.*001*).

Recently, lncRNA ANRIL was found to promote tumour cell proliferation and decrease cell apoptosis in a variety of cancers^25,29^. Moreover, based on the association of lncRNA ANRIL with a more malignant cancer phenotype in OSCC, we hypothesized that lncRNA ANRIL was involved in OSCC cell proliferation and sensitivity to cisplatin. Therefore, we silenced its expression via RNAi to investigate the effect of lncRNA ANRIL on tumour cell growth. QPCR analysis of lncRNA ANRIL levels was performed 48 hours after transfection. lncRNA ANRIL expression was significantly down-regulated in si-ANRIL-transfected HSC3, OSCC3 and SCC4 cells when compared with control cells (si-NC; Fig. 5a). Meanwhile, we assessed the effects of ANRIL on OSCC cell proliferation *in vitro* by CCK-8 assay and colony formation assays. Consistent with our hypothesis, knockdown of lncRNA ANRIL in HSC3, OSCC3 and SCC4 obviously inhibited cell proliferation compared to that in tumour cells transfected with si-NC (Fig. 5b). In addition, there were fewer colonies of HSC3, OSCC3 and SCC4 cells transfected with si-ANRIL than negative control treatment groups (Fig. 5c). Cells transfected with si-ANRIL had increased cell apoptosis according to the result of flow cytometric analysis (Fig. 5d,e). These results suggest that down-regulated expression of lncRNA ANRIL inhibits OSCC cell proliferation and induces cell apoptosis *in vitro*.

To further examine the effect of lncRNA ANRIL knockdown on the sensitivity of OSCC cells to cisplatin, the level of apoptosis in cells transfected with si-ANRIL or si-NC before the cisplatin treatment was detected by flow cytometry. The results revealed that lncRNA ANRIL knockdown sensitized HSC3, OSCC3 and SCC4 cells to cisplatin-induced cell death in comparison with that in control cells (Fig. 5d,e). These data indicate that lncRNA ANRIL can promote the resistance of OSCC cells to cisplatin.

**Figure 6:**
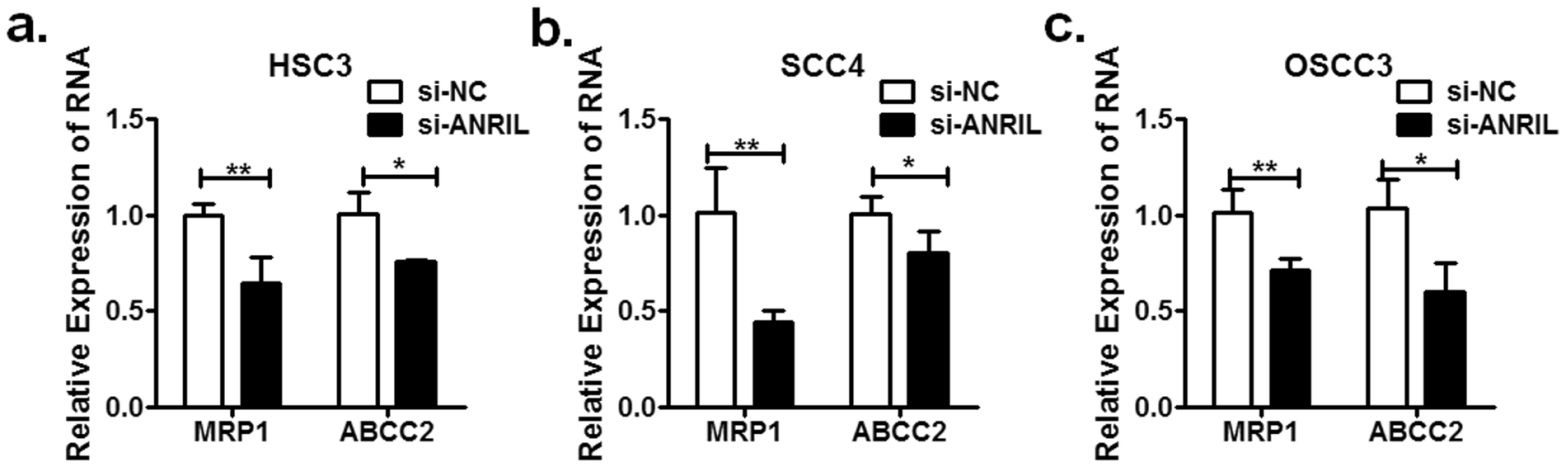
MRP1 and ABCC2 expression in OSCC cells with ARNIL knocked down was determined by Q-PCR. Values represent the mean ± SD from three independent experiments (*P < *0*.*05*, **P < *0*.*01*, ***P < *0*.*001*).

Induction of chemoresistance in many cellular settings relies on increasing efflux of the drug, which is triggered by the stimulation of ATP-binding cassette (ABC) transporter proteins^30^. Accordingly, knockdown of lncRNA ANRIL in HSC3, OSCC3 and SCC4 cells suppressed MRP1 and ABCC2 expression, which may be responsible for the decreasing efflux of cisplatin (Fig. 6). Altogether, these observations indicate that lncRNA ANRIL can promote the resistance of OSCC cells to cisplatin.

### LncRNA ANRIL knockdown overcomes MK-induced cisplatin resistance via activation of the caspase-3-dependent apoptotic pathway

**Figure 7:**
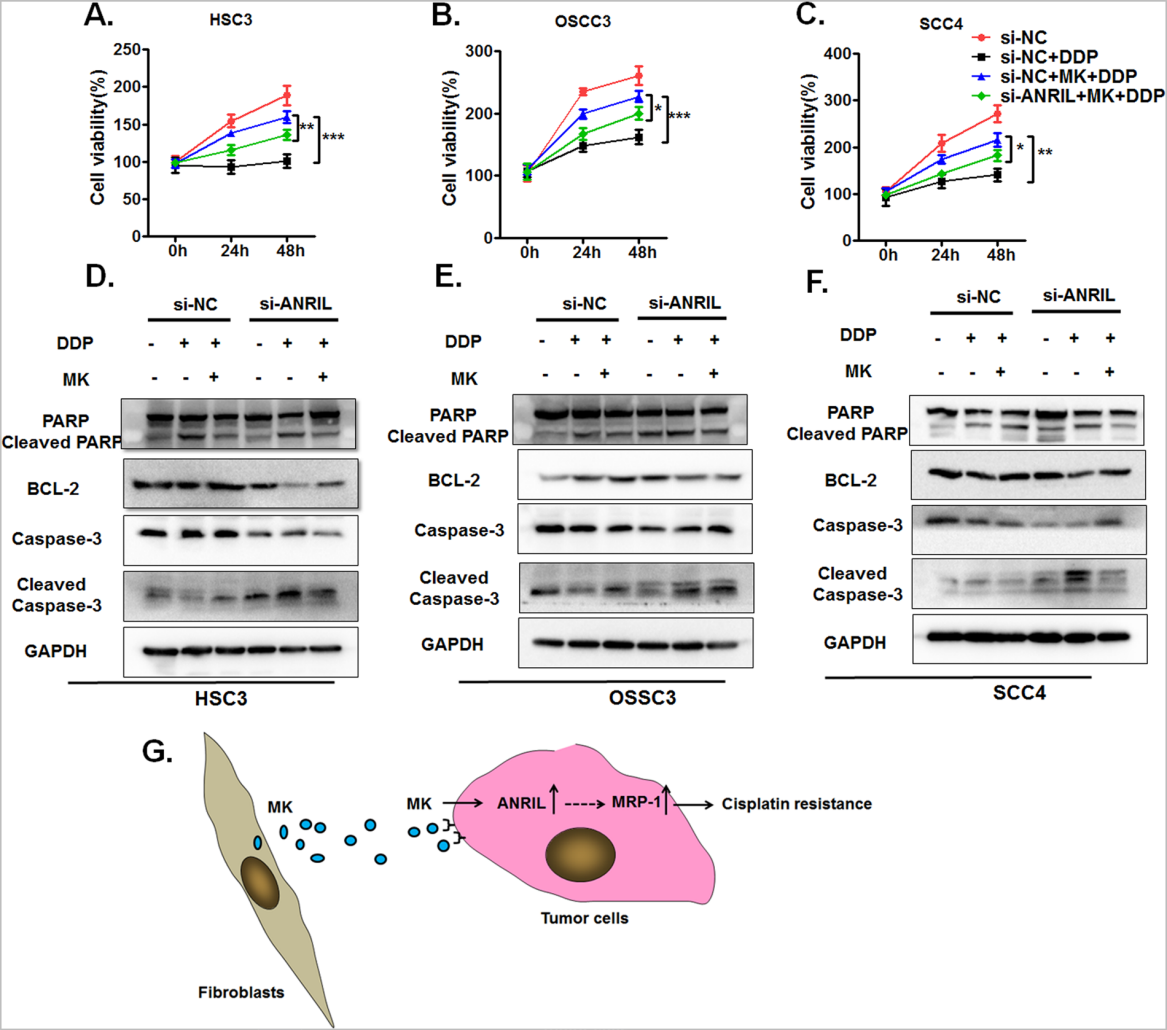
ANRIL knockdown overcomes MK-induced cisplatin resistance via activation of caspase-3-dependent apoptosis. The effect of MK on the cell viability of cisplatin-treated HSC3 (**a**), OSCC3 (**b**) and SCC4 (**c**) cells transfected with either si-NC or si-ANRIL was determined by CCK-8 assays. Values represent the mean ± SD from three independent experiments (*P < *0*.*05*, **P < *0*.*01*, ***P < *0*.*001*). The expression of PARP, Bcl-2, cleaved caspase-3 and caspase-3 in siNC- or si-ARNIL-transfected HSC3 (**d**), OSCC3 (**e**) and SCC4 (**f**) cell lines with either treatment of exogenous MK (100 ng/ml) or without MK was analysed by western blot. A representative experiment of four is shown. (**g**) A suggested mechanism for the CAF-derived MK-mediated enhancement of tumour cell resistance to cisplatin.

To further investigate whether MK-induced cisplatin resistance was related to the up-regulation of lncRNA ANRIL in OSCC cells, lncRNA ANRIL expression was silenced in OSCC cells that were treated with MK. In the group of OSCC cells transfected with si-NC, the same results were observed by means of CCK-8 assay at each measured time (Fig. 7a–c). Previous studies have indicated that lncRNA ANRIL could contribute to cancer cell proliferation by inhibiting cell apoptosis^25^. Therefore, we further studied whether knockout lncRNA ANRIL could eliminate the midkine-induced resistance to cisplatin-induced cell death in OSCC lines through the apoptotic signalling pathway. We determined the levels of apoptosis-related proteins, including PARP, cleaved PARP, Bcl-2, caspase-3, and cleaved caspase-3, in si-NC- or si-ANRIL-transfected OSCC cell lines with MK treatment (100 ng/ml) for 24 h by western blotting. The results of the western blot assays showed that cleaved PARP and cleaved caspase-3 expression was obviously increased in the si-ANRIL group, the si-ANRIL group with DDP added for 48 h, and the si-ANRIL group that was pre-treated with MK for 24 h before the addition of DDP for 48 h compared with the corresponding treatment group in OSCC cell lines. Moreover, cleaved PARP and cleaved caspase-3 were significantly reduced in the group that had MK and DDP added compared to the group that had DDP added but not MK. In contrast to these findings, Bcl-2 and caspase-3 expression correspondingly decreased in the si-ANRIL group, the si-ANRIL group with DDP added, and the si-ANRIL group that was pre-treated with MK for 24 h before the addition of DDP compared with the corresponding treatment group in OSCC cell lines. Meanwhile, Bcl-2 and caspase-3 expression correspondingly increased in the group that had MK and DDP added compared to the group that had DDP added but not MK. (Fig. 7d–f). The relative expression of these protein confirmed the results above (Supplementary Fig. S5). These data suggest that silencing lncRNA ANRIL enhanced OSCC cell sensitivity to cisplatin by the apoptotic pathway.

Taken together, these findings show that CAF-derived MK acts on tumour cells by paracrine action, increasing lncRNA ANRIL expression in tumour cells and thus promoting the up-regulation of ABC family proteins MRP1 and ABCC2, which ultimately results in tumour cell resistance to cisplatin. These findings provide new insights into the source of MK in tumour tissues, which may serve as a novel therapeutic approach for cancer.

## Discussion

Recently, emerging evidence has characterized cancer-associated fibroblasts (CAFs), a component of the tumour microenvironment, and shown the important roles they play in chemoresistance, which has provided new insights into the biology of carcinomas^31^. The intercellular crosstalk between CAFs and tumour cells influences drug resistance by promoting the secretion of survival factors. For example, increased secretion of IL-11 from CAFs treated with cisplatin mediates lung adenocarcinoma cell chemoresistance through paracrine signalling of the IL-11/IL-11R/STAT3 pathway^32^. In our study, we demonstrated that conditioned medium obtained from CAFs increases the viability of cisplatin-treated tumour cells. In addition, we detected a notable enrichment of MK in the CAF-conditioned medium. Although other studies have revealed that midkine can exert a survival function in cancer cells and promote tumour progression^6,7,33,34^; these studies merely focused on the effect of MK expression in tumour cells on chemoresistance^9^. There is little research about the effect of CAF-secreted MK on tumour cell resistance to cisplatin. Here, we are the first to describe that CAFs taken from tumour tissues can secrete large amounts of MK and that MK enhanced tumour cell resistance to cisplatin by a paracrine mode of action.

Accumulating evidence shows that numerous lncRNAs are well characterized and exert significant functions in tumourigenesis, suggesting that they could provide new insights into the biology of this disease. Among these lncRNAs, metastasis-associated lung adenocarcinoma transcript 1 (MALAT1) is a highly conserved nuclear lncRNA that promotes tumour cell migration and cell growth by regulating gene expression, including the regulation of metastasis-associated transcripts, and can serve as a predictive marker for the development of metastasis in lung cancer^35^. In addition, up-regulation of MVIH expression in HCC is correlated with microvascular invasion. MVIH associates with phosphoglycerate kinase 1 (PGK1) and inhibits its secretion, which promotes tumour growth and intrahepatic metastasis by contributing to active angiogenesis both *in vitro* and *in vivo* ^36^, but the expression of lncRNA ANRIL in OSCC and its association with cisplatin resistance are still not well documented. In this study, we first illustrated that the expression of lncRNA ANRIL is significantly up-regulated in OSCC tissues and OSCC cell lines and is correlated with high TNM stage and lymph node metastasis (LNM). Increased lncRNA ANRIL expression appears to be a significant, independent predictive factor for patients with advanced OSCC.

Moreover, knockdown of lncRNA ANRIL expression in OSCC cells partly abrogated MK-induced cisplatin resistance. We also found that silencing lncRNA ANRIL inhibited cell proliferation and promoted cell apoptosis. These findings suggest that lncRNA ANRIL plays a direct role in cell proliferation and OSCC progression and that it is associated with drug resistance, which could allow it to serve as a useful, novel prognostic or therapeutic target for OSCC. There are numerous studies that suggest lncRNAs play roles in multiple tumours, including NSCLC, by binding with certain proteins and influencing their downstream target genes^37^. It has been reported that ANRIL is involved in cancer cell proliferation by silencing p15/INK4 expression^23^. Here, we found that inhibition of lncRNA ANRIL expression repressed MRP1 and ABCC2 expression in OSCC cell lines. Furthermore, knockdown of lncRNA ANRIL influenced MK-induced cisplatin resistance in OSCC cells via increasing levels of cleaved caspase-3 and suppressing Bcl-2 expression at the protein level. As shown above, the contribution of lncRNA ANRIL to OSCC cell chemoresistance could be dependent on its regulation of MRP1 and ABCC2 expression. Our investigation underlined the relationship between CAF-derived MK, ANRIL and chemoresistance.

In summary, cancer-associated fibroblasts confer chemoresistance to tumour cells. CAF-secreted MK decreased cisplatin-induced apoptosis and promoted tumour cell drug resistance. Furthermore, MK enhanced tumour cell resistance to cisplatin by inducing lncRNA ANRIL expression and increasing anti-apoptotic protein Bcl-2 expression in cancer cells. These findings demonstrate that MK secreted by CAFs plays a role in cisplatin-based chemoresistance. Research into the mechanism by which this occurs will be essential in future projects.

## Materials and Methods

### Ethics statement

All aspects of this study were approved by the Research Ethics Committee of Nanjing Stomatology Hospital Affiliated to Nanjing University, and were carried out in accordance with the approved guidelines. Written informed consent was obtained from all patients and the Research Ethics Committee of the Nanjing Stomatology Hospital Affiliated to Nanjing University. Informed consent was obtained from all subjects enrolled in the studies that provided the samples. All these specimens were handled and anonymized according to ethical and legal standards.

### Tumour tissue specimens

We obtained 60 paired oral squamous cell cancer tissues and adjacent non-tumour tissues from patients who were diagnosed with primary OSCC by the Department of Pathology at Nanjing Stomatology Hospital, and ethical approval for the use of oral squamous cell carcinoma specimens for this study was obtained from the Research Ethics Committee of Nanjing Stomatology Hospital. Among all the subjects, those who were pregnant or lactating, and patients who were diagnosed with other malignant diseases or some autoimmune diseases (e.g., rheumatoid arthritis, systemic lupus erythematous, or diabetes) were excluded from our experimental group, and all the patients had no long history of alcohol abuse. None of the patients underwent preoperative chemotherapy and/or radiotherapy. In this study, all the OSCC tissues were evaluated according to WHO classifications by two pathologists.

### Cell lines and cell culture

The normal human keratinocyte line HaCaT and the human oral squamous cancer cell lines OSCC3, SCC4, HSC3 and CAL27 were cultured in Dulbecco’s modified Eagle’s medium; the human ovarian cancer cell line A2780 was cultured in Dulbecco’s modified Eagle medium/nutrient mixture F-12 (Life Technologies, USA); the lung cancer cell line A549 was cultured in RPMI medium 1640 supplemented with 10% FBS, 100 U/ml penicillin, and 100 µg/ml streptomycin (Gibco, USA). All cell lines were incubated in 5% CO2 at 37 °C conditions.

### Cell proliferation assay

HSC3, OSCC3, SCC4 cells were seeded at a density of 5000 cells/well in 96-well plates (100 µl/well) for 24 h before treatment with exogenous midkine for a specific amount of time with CAF conditioned medium alone or in combination with anti-MK antibody. A2780 and A549 cells were plated at a density of 3000 cells/well in 96-well plates for 24 h before treatment with exogenous midkine for a specific amount of time with CAF conditioned medium alone or in combination with anti-MK antibody^13^ (20 µg/mL, Santa Cruz, CA, USA). Cell viability was determined after treatment with cisplatin (6 µM, 5 µM, 9 µM, 7.7 µM and 4.8 µM) for 48 h by a Cell Counting Kit-8 (CCK8, Dojin Laboratories, Kumamoto, Japan) according to the manufacturer’s instructions, and the absorbance of each well was measured at 450 nm with a microtiter plate reader (Hynergy HT, BioTek, Winooski, VT). The HSC3, OSCC3 and SCC4 cells transfected with si-NC or si-ANRIL (5000 cells/well) were grown in 96-well plates. Cell viability was assessed every 24 hours following the manufacturer’s protocol. Cell viability was calculated as the ratio of treated cells to untreated cells.

### Colony formation assay

Cells transfected with si-NC or si-ANRIL were harvested 48 hours after transfection by trypsinization and seeded into 6-well plates (2000 cells per well), after which they were cultured for two weeks. Visible colonies were fixed with methanol and stained with 0.1% crystal violet. Colonies with more than 50 cells were counted.

### Cell transfection

The small interfering RNA ANRIL (si-ANRIL) used for silencing human ANRIL and small interfering RNA negative control (si-NC) were obtained from RiboBio. Either the si-ANRIL plasmid or the si-NC plasmid was transfected into HSC3, OSCC3 and SCC4 cells. Cells were grown in 6-well plates to 60% confluence and transfected using Lipofectamine 2000 (Invitrogen) according to the manufacturer’s instructions, and the final concentration of siRNAs was 60 nM. At 48 h after transfection, cells were harvested for qPCR or western blot analysis. The siANRIL sequences used in this study were as follows: 5′-GGUCAUCUCAUUGCUCUAUTT-3′.

### Reverse transcription and real-time quantitative PCR analysis

Total RNA was isolated from cells or tissues using TRIzol reagent (Invitrogen) according to the manufacturer’s instructions. The conditions used for reverse transcription of RNA to cDNA were 42 °C for 5 min, 99 °C for 20 min, and 4 °C for 5 min. Quantitative real-time RT-PCR (qRT-PCR) was performed using SYBR Green PCR Master Mix (Bio RAD) according to the manufacturer’s instructions. The qPCR assays were carried out on a StepOne Plus analyser, and data were collected from this instrument. Relative gene expression was calculated with the 2^−∆∆CT^ formula, and results were normalized to the expression of GAPDH. All the primer sequences are shown in Supplementary Table 1.

### Flow cytometric assay

HSC3, OSCC3 and SCC4 were seeded at the density of 5 × 10^5^ cells/well in 12-well plates for 24 h before cells were transfected with either si-NC or si-ANRIL. After transfection with siRNA, cells were treated with a corresponding concentration of cisplatin and exogenous MK (100 ng/mL) for 48 h. Cells were harvested by trypsinization, centrifuged to remove the medium, washed once with binding buffer (10 mM HEPES, 140 mM NaCl, and 2.5 mM CaCl_2_ in aquadest), and then stained with FITC-Annexin V and PI (BD, San Diego, CA, USA) according to the manufacturer’s instructions. Cells were detected by flow cytometry (FACScan; Becton Dickinson, MountainView, CA, USA). Viable cells were negative for both PI and Annexin V; apoptotic cells were positive for Annexin V and negative for PI; and late apoptotic, dead cells displayed both high Annexin V and PI labelling. Nonviable cells that underwent necrosis were positive for PI and negative for Annexin V. The apoptotic rate was compared with the control treatment from each experiment.

### Western blot analysis

HSC3, OSCC3, and SCC4 cells were lysed in buffer containing 50 mmol/L Tris-HCl (pH 8.0), 150 mmol/L NaCl, 0.02% NaN_3_, 0.1% SDS, 100 mg/L phenylmethylsulfonylfluoride, 1 mg/L aprotinin, and 1% Triton. Cell extracts were separated by SDS-PAGE and transferred onto PVDF membranes. The membranes were blocked for 1 h in TBST (10 mmol/L Tris-HCl (pH 7.4), 150 mmol/L NaCl, 0.05% Tween-20) containing 5% bovine serum albumin (BSA); incubated with primary antibodies against cleaved caspase-3 (Cell Signalling Technology (CST), Danvers, MA, USA), Bcl-2 (CST), PARP (CST), caspase-3 (CST), and GAPDH (CST) at 4 °C overnight; and then incubated with secondary antibodies. Bands were visualized with an enhanced chemiluminescence reaction (Millipore Corp.). GAPDH was used as the loading control. Protein bands were captured and analysed using LANE 1D software (Sage Creation Science Co, Beijing).

### Statistics

Statistical analyses were performed using Graph Pad Prism (San Diego, CA). Two-tailed Student’s t tests were used for the comparison of experimental groups. Statistical significance was defined at equal to or more than a 95% confidence interval or *P* ≤ *0*.*05*. Experiments presented are representative of 3 repeated experiments. Data are presented as the mean ± standard deviation.

## Electronic supplementary material

Supplementary information
