# Supplementary material for: Midkine derived from cancer-associated fibroblasts promotes cisplatin-resistance via up-regulation of the expression of lncRNA ANRIL in tumour cells
Source: Sci Rep. 2017 Nov 24;7:16231. doi: 10.1038/s41598-017-13431-y (PMC5701200; doi:10.1038/s41598-017-13431-y)
Supplement: Supplementary file 1 — Supplementary information [file 41598_2017_13431_MOESM1_ESM.pdf]

Midkine derived from cancer-associated fibroblasts promotes cisplatin-resistance via up-regulation of the expression of lncRNA ANRIL in tumour cells

Dongya Zhang[1,#], Liang Ding[1,#], Yi Li[1], Jing Ren[1], Guoping Shi[1], Yong Wang[1], Shuli Zhao[2*], Yanhong Ni[1*], Yayi Hou[1*]

[1]The State Key Laboratory of Pharmaceutical Biotechnology, Division of Immunology and Hospital of Stomatology, Medical School, Nanjing University, Nanjing, China.

[2] Nanjing First Hospital, Nanjing Medical University, Nanjing, China.

# Contributed equally to this work

***Corresponding author**: Yayi Hou, The State Key Laboratory of Pharmaceutical Biotechnology, Division of Immunology, Medical School, Nanjing University, Nanjing, 210093, China. Tel/fax: +86-25-83686341; Email: yayihou@nju.edu.cn

Or Yanhong Ni, Central Laboratory, Hospital of Stomatology, Medical School, Nanjing University, Nanjing, 210008, China. Tel.: +86-025-83620140; Email: niyanhong12@163.com

Or Dr Shuli Zhao, Nanjing First Hospital, Nanjing Medical University, Nanjing, China; No. 68 Chang Le Road, Nanjing, 210006, China. Email: shulizhao79@163.com

Supplementary Table S1

Table S1. Primers used for real-time RT-PCR

Gene	Sequence	
	Forwards primers	Reverse primers
ANRIL	TGCTCTATCCGCCAATCAGG	GGGCCTCAGTGGCACATACC
MRP1	AAGGAGGTACTAGGTGGGCTT	CCAGTAGGACCCTTCGAGC
ABCC2	TCTCTCGATACTCTGTGGCAC	CTGGAATCCGTAGGAGATGAAGA
GAPDH	GGAGCGAGATCCCTCCAAAAT	GGCTGTTGTCATACTTCTCATGG

Supplementary Figure S1

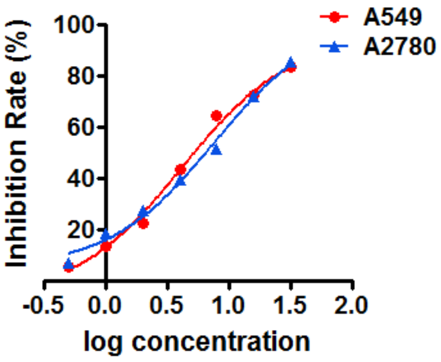

	IC50 (95% CI)
A549	4.791 (2.419-7.110)
A2780	7.706 (3.974-14.95)

Suppl.Figure S1.Inhibition rateand inhibition concentration at 50% (IC50) values of DDP for A549 and A2780. A549 and A2780 were seeded into 96-well plate (3000 cells/well) for 24h before treating different concentration of DDP. Cell viability was determined by CCK-8 Kit after culture 48h. Data are representative of three independent experiments. IC50 of DDP for A549 and A2780 was calculated by GraphPad Prism.

Supplementary Figure S2

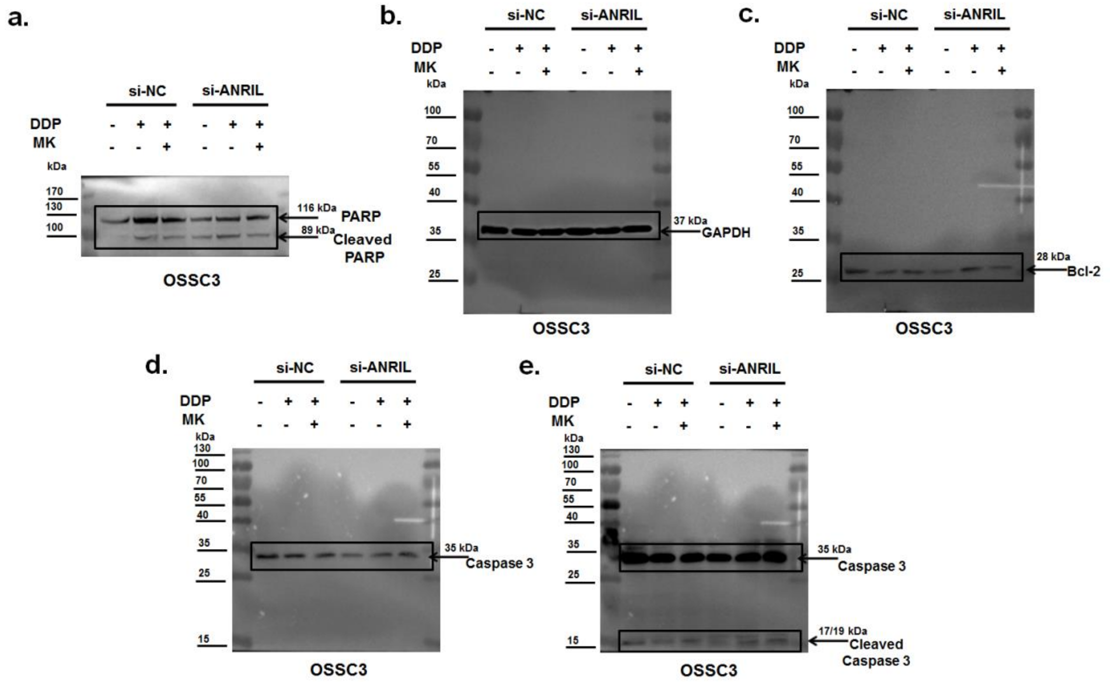

Suppl. Figure S2. Expression of PARP, Cleaved PARP, Caspase-3, cleaved caspase-3, Bcl-2 in OSCC3 cells.(a) Cleaved PARP was increased in si-ANRIL OSCC3 cells,

and more increased in si-ANRIL OSCC3 cells treated with DPP, compared to si-NC OSCC3 cells. (b) GAPDH was used as internal control. (c) Bcl-2 expression was more reduced in si-ANRIL OSCC3 cells treated with DDP (5μM) compared to addition of MK in si-ANRIL OSCC3 cells treated with DDP. (d) Caspase-3 expression was more decreased in si-ANRILOSCC3 cells than si-NC OSCC3 cells, and MK increased caspase-3 expression. (e) Cleaved caspase-3 was more increased in si-ANRIL OSCC3 cells treated with DDP (5μM) compared to addition of MK in si-ANRIL OSCC3 cells treated with DDP. The protein level of PARP, cleaved PARP, caspase-3, cleaved caspase-3, Bcl-2 in OSCC3 cells wasmeasured by western blotting.The gels were rununder the same experimental conditions and the full-length gels/blots were displayed. Thearea encircled by the solid line was cropped for final display (Figure 7e).

Supplementary Figure S3

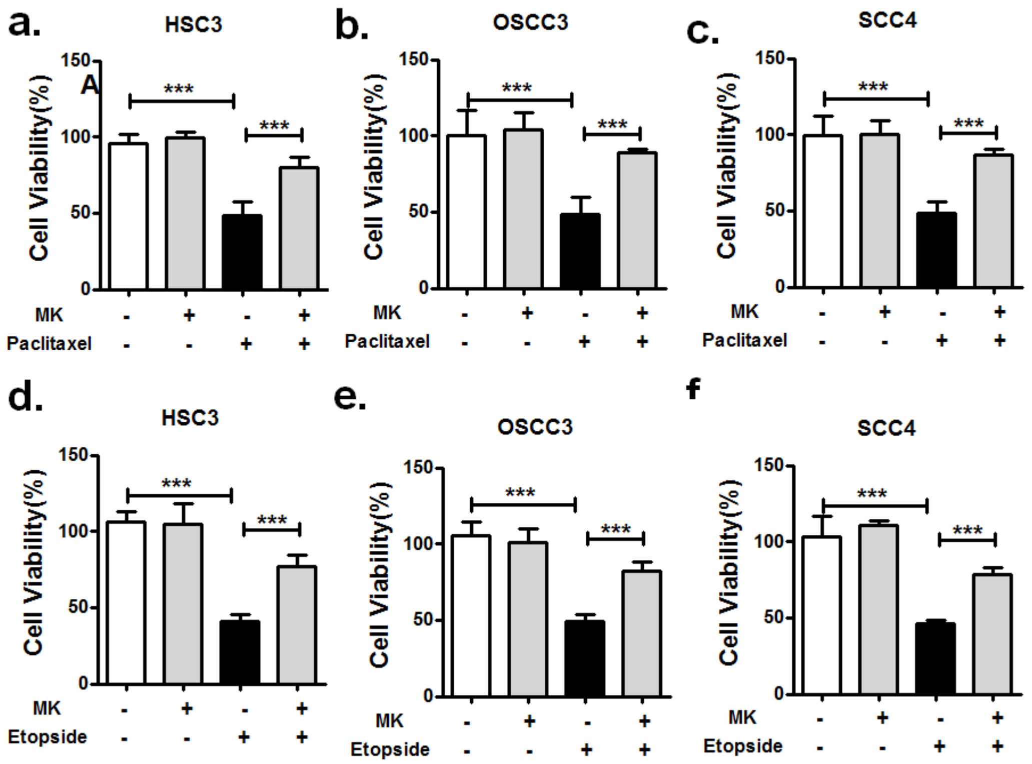

Suppl. Figure S3.MK increases the survival of Paclitaxel or Etopside-treated tumor cells in vitro. Effect of Paclitaxel or Etopside on tumor cells with pretreatment of MK is determined by means of cell viability assay via CCK-8 kit (*P<0.05, **P<0.01, ***P<0.001). (a-e) HSC3, OSCC3, SCC4 were treated with exogenous MK (100ng/mL) for 48h before the treatment with corresponding concentration of Paclitaxel (HSC3 20nM, OSCC3 15nM, SCC4 30nM) or Etopside (HSC3 50μM, OSCC3 40μM, SCC4 55μM) for 48h. Data are representative of three independent experiments.

Supplementary Figure S4

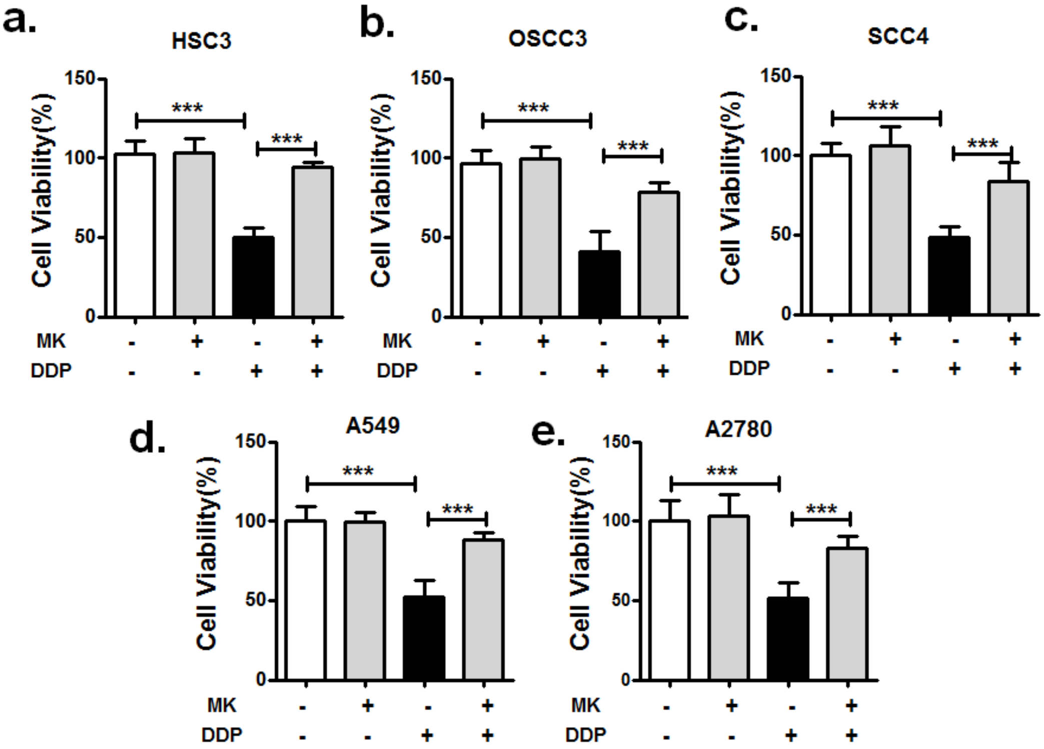

Suppl. Figure S4.MK increases the survival of cisplatin-treated tumor cells in vitro.Effect of cisplatin on tumor cells with pretreatment of MK is determined by means of cell viability assay via CCK-8 kit (*P<0.05, **P<0.01, ***P<0.001). (a-e) HSC3, OSCC3, SCC4, A549 and A2780 were treated with exogenous MK(100ng/mL) for 48h before the treatment with corresponding concentration of cisplatin (HSC3 6μM, OSCC3 5μM, SCC4 9μM, A2780 7.7μM and A549 4.8μM) for 48h. Data are representative of three independent experiments.

Supplementary Figure S5

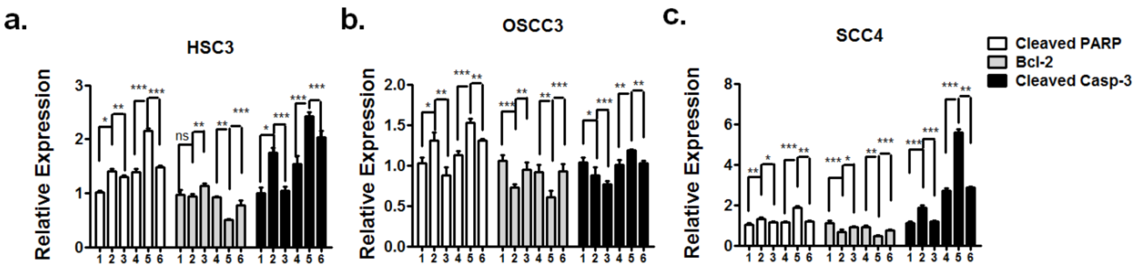

Suppl. Figure S5.ANRIL knockdown overcome MK-induced cisplatin resistance via activation caspase-3-dependent apoptosis. (a-c) The relative expression of cleaved PARP, Bcl-2 and Cleaved Caspase-3 to GAPDH was calculated. Numbers were used to represent for corresponding experiment groups in Figure 7d-e.

Fig. 7D

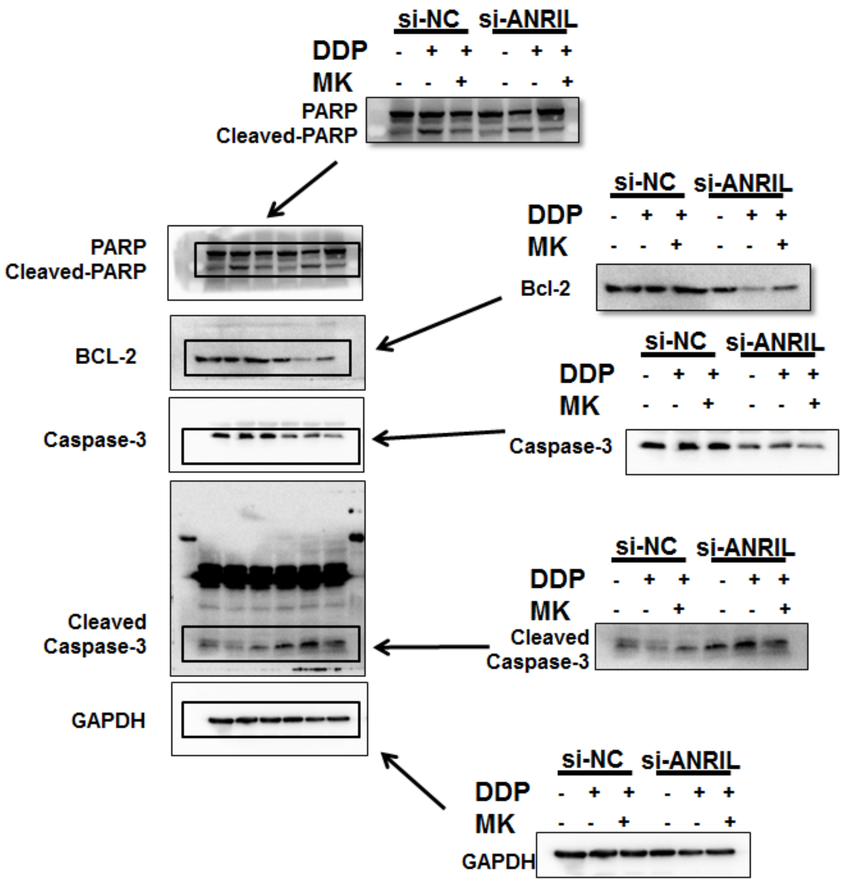
si-NC
si-ANRIL
DDP
MK
PARP
Cleaved-PARP
BCL-2
Caspase-3
Cleaved
Caspase-3
GAPDH
Bcl-2

Fig. 7E

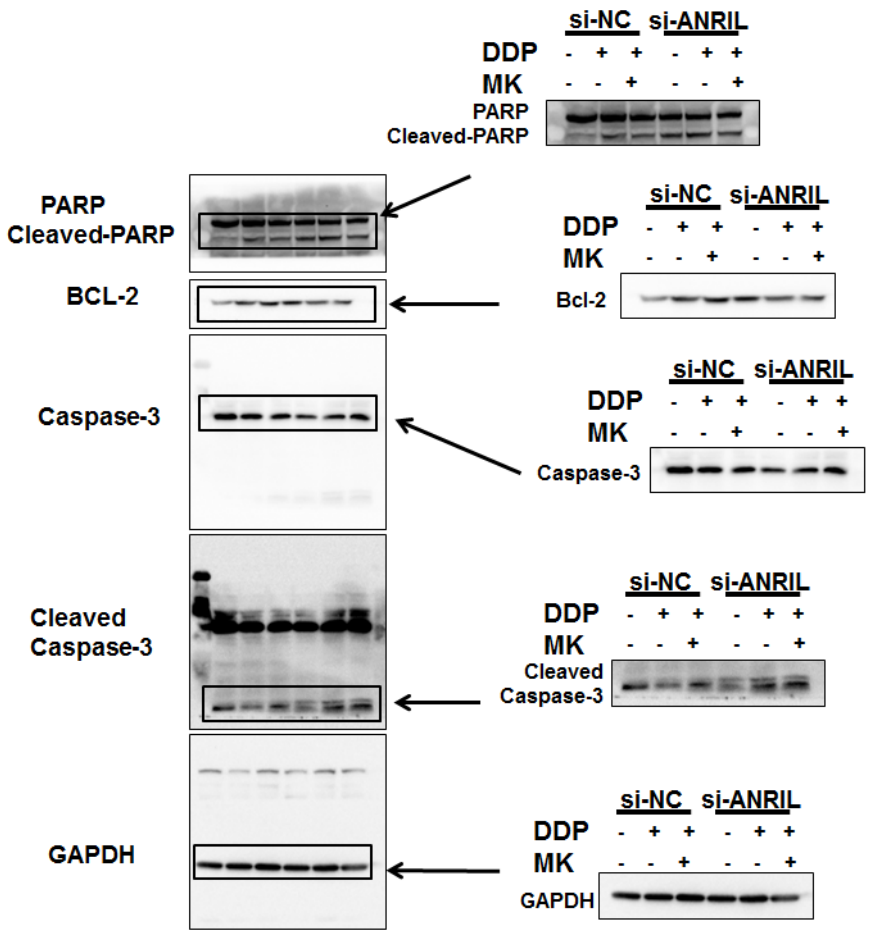

Fig. 7F

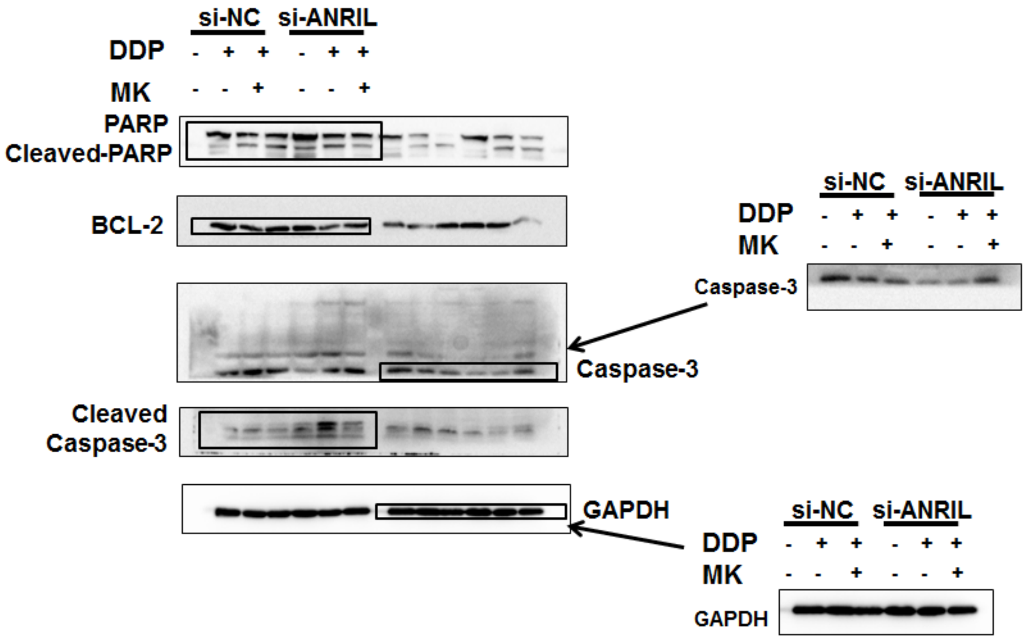

Fig.（7D-7F). Expression of PARP, Cleaved PARP, Caspase-3, cleaved caspase-3,

Bcl-2 in HSC3, OSCC3, SCC4 cells. The gels were rununder the same experimental conditions and the full-length gels/blots were displayed. Thearea encircled by the solid line was cropped for final display (Figure 7).
